# Evaluation of phytochemical and superoxide dismutase activities of *Enhalus acoroides* (L.f.) Royle from coastal waters of North Sulawesi, Indonesia

**DOI:** 10.14202/vetworld.2020.676-680

**Published:** 2020-04-13

**Authors:** Febry S. I. Menajang, Mohammad Mahmudi, Uun Yanuhar, Endang Yuli Herawati

**Affiliations:** 1 Doctoral Program, Faculty of Fisheries and Marine Science, Brawijaya University, Malang 65145, East Java, Indonesia; 2 Department of Aquatic Resource Management, Faculty of Fisheries and Marine Science, Sam Ratulangi University, Manado, 95115, North Sulawesi, Indonesia; 3 Department of Aquatic Resource Management, Faculty of Fisheries and Marine Science, Brawijaya University, Malang 65145, East Java, Indonesia

**Keywords:** *Enhalus acoroides* (L.f.) Royle, phytochemical, superoxide dismutase

## Abstract

**Background and Aim:**

Seagrasses are an excellent and potential bioresource to discover new natural bioactive compounds such as antioxidants that have beneficial effects on health. Natural antioxidants have many functions in biological systems, primarily for defense against oxidation which produces free radicals in food, chemicals, and living systems. This study aimed to discover new natural antioxidant agents, *Enhalus acoroides* (L.f.) Royle was evaluated for phytochemical constituents and the antioxidant activity against superoxide dismutase (SOD) was assessed.

**Materials and Methods:**

Sample specimens of *E. acoroides* (L.f.) Royle collected from two different areas, Manembo-Nembo, Bitung (SG-A) and Bahoi, Likupang Barat (SG-B) waters, were extracted with methanol and solutions were prepared in a concentration series.

**Results:**

Extracts of the seagrass *E. acoroides* (L.f.) Royle cultivated in different areas have different phytochemical constituents and SOD activities. The secondary metabolites of phenols, flavonoids, and steroids contained in the ethyl acetic extracts of *E. acoroides* were linearly correlated with their antioxidant activity, whichexhibited an IC_50_ of 7 ppm.

**Conclusion:**

*E. acoroides* (L.f.) Royle samples cultivated in the two areas contained different phytochemical constituent profiles, indicating an effect of environmental factors, and both can be used as potential natural sources of antioxidant compounds.

## Introduction

Free radicals are highly reactive compounds produced in our body through daily metabolic processes [1]. Antioxidants in biological systems have many functions, including protection from oxidative damage and in major signaling pathways of the cell. The main action of antioxidants in cells is to prevent damage caused by the action of reactive oxygen species (ROS) [2]. Natural antioxidants have many functions in biological systems primarily for defense against oxidation, which produces free radicals in food, chemicals, and living systems [3]. Because of carcinogenicity, synthetic compounds have a deficiency in natural antioxidants. Natural antioxidants have an important role in the mechanism of antioxidant defense in biological systems and act as an antidote to free radicals [4]. To prevent damage from free radicals, living organisms, including humans, have an immune system that can produce antioxidants naturally from the body, namely, endogenous antioxidants [5]. In addition, antioxidants can also be obtained from food intake, i.e. exogenous antioxidants. Thus, many natural antioxidants have been isolated from various natural resources such as vegetable oil, cereal crops, vegetables, spices, and herbs [6]. In sea plants, there are two good groups as sources of antioxidants, namely, seaweeds and seagrasses [5]. Seagrasses are a group of around 60 species of flowering plants growing in the sea and form the most extensive and productive coastal system in the world [7].

In traditional medicine, *Enhalus acoroides* (L.f.) Royle has been used for various repair purposes, for example, for the treatment of fever and skin diseases [8], muscle aches [9], wounds [10], stomach problems [11], drugs for stings from various types of rays [12], and as sedatives for babies [13]. *E. acoroides* (L.f.) Royle produces antimicrobial compounds that can act to reduce or control microbial growth and many studies describe antibacterial [14], antifungal [15], antiviral [16], anti-inflammatory [17], antidiabetic [18], and antioxidant [19] activities. Seagrasses, including *E. acoroides* (L.f.) Royle, are easily found in Indonesia; the species can be easily identified because it has distinct morphologies. *E. acoroides* (L.f.) Royle roots, stems, and leaves are larger than those of other seagrass species found in Indonesia [20]. Seagrasses are a functional group of flowering plants rooted in the world’s coastal seas, known for their secondary metabolites [21]. Seagrasses comprise 49 species from 12 genera distributed throughout the world. *E. acoroides* (L.f.) Royle belongs to *Enhalu*s, a monotypic marine genus in the family Hydrocharitaceae, which is widespread along the coast of the Indian Ocean and the tropical Pacific West [22].

*E. acoroides* (L.f.) Royle breed and develop several strategies to survive in an environment with a salt concentration of about 200 mM NaCl or more. When such plants grow in very high saline conditions, it is hypothesized that they can have rare and new activities, which are not reported in their terrestrial relatives [23]. Humans, especially coastal residents, usually consume *E. acoroides* (L.f.) Royle as food [24].

This study aimed to discover new natural antioxidant agents, *Enhalus acoroides* (L.f.) Royle was evaluated for phytochemical constituents and the antioxidant activity against superoxide dismutase (SOD) was assessed.

## Materials and Methods

### Ethical approval

Ethics approval was not required for this study.

### Chemicals and reagents

The distillated organic solvents of *n*-hexane, ethyl acetic, and methanol were used for sample extraction. For the antioxidant assay, SOD reagents nitro blue tetrazolium (NBT), riboflavin, N,N,N’,N’-tetramethyl ethylenediamine (TEMED), and PBS were purchased from Amresco. The thin-layer chromatography (TLC) spray reagent, 10% sulfuric acid in ethanol, used in this study was purchased from Merck and Sigma-Aldrich. Kiesel G 60 silica gel resins and ODS columns of LiChroprep RP-18 (Merck, Darmstadt, Germany) were used for column chromatography. TLC analysis was performed using Kiesel gel 60 F_254_ and RP-18 F_254S_ (Merck). Deuterated solvents were purchased from Merck Co., Ltd., and Sigma-Aldrich Co., Ltd. (St. Louis, MO, USA).

### Instruments

Nuclear magnetic resonance (NMR) spectra were recorded on a 500 MHz Fourier transform (FT)-NMR spectrometer (Varian ECA 500 JOEL, Japan). Infrared (IR) spectra were obtained using a PerkinElmer Spectrum One FT-IR spectrometer (Buckinghamshire, England). Mass spectra were obtained using an electrospray ionization mass spectrometry (ESI-MS) (ultra-performance liquid chromatography MS/MS TQD type, Waters). 2,2-diphenyl-1-picrylhydrazyl and SOD assays were measured on a Biochrom Ez Read 400 ELISA Reader.

### Plant material collection and determination

The seagrass *E. acoroides* (L.f.) Royle was cultivated and collected in May 2016 from Manembo-Nembo, Bitung (SG-A) and Bahoi, Likupang Barat, Manado (SG-B), North Sulawesi, Indonesia. The specimen was deposited and identified at the Laboratory of Plant Taxonomy, Department of Biology, Faculty of Mathematics and Natural Sciences, Padjadjaran University, Bandung, Indonesia.

### Preparation of extract

After collection, the fresh plants of *E. acoroides* (L.f.) Royle were washed well and cut into small-sized pieces. The samples were extracted with methanolfor 3days and subsequently partitioned using water-*n*-hexane and water-ethyl acetate to yield concentrated extracts, as shown in Table-1. The obtained extract was filtered, concentrated in vacuum, and divided into a series of concentrations for phytochemical screening and SOD antioxidant activity assessment.

**Table-1:** Data of weight extracts of specimen.

Samples	Extracts weight (g)			

	MeOH	*n*-hexane	EtOAc	H_2_O
SG-A (4.13 kg)	101.34	20.51	40.67	16.89
SG-B (2.23 kg)	98.25	15.34	38.88	20.67

### Preliminary phytochemical screening

Screening for secondary metabolites such as alkaloids, terpenoids, and flavonoids was performed using methanol, *n*-hexane, ethyl acetate, and water extracts of vegetables and fruits as described previously [25,26].

### TLC analysis

The chemical constituent profile of the extract and pure active compounds of the seagrass *E. acoroides* (L.f.) Royle cultivated in different areas was analyzed using a combination of TLC on Silica 60 G_F254_ and ODS RP-18 plates eluted with a combination of organic solvent and H_2_O. Chemical compounds on plates were identified and observed under ultraviolet (UV) lamps at 254 and 365nm and through the reaction to the specific spraying reagent of H_2_SO_4_ in 10% EtOH [25,26].

### SOD antioxidant activity assay

The SOD-mimic activity of compound 1 was evaluated using an indirect method of riboflavin photoreduction as described previously [27]. The method involves the competitive reaction between the complex and reduced NBT for O_2_% − generated by riboflavin under illumination at room temperature (25°C). The sample mixture (240 µL) contained the complex (11 different concentrations), 6 µM riboflavin, 0.8 µM of *N*,*N*,*N’*,*N’*-tetramethylethylenediamine (TMEDA) in 0.016 M phosphate buffer (pH7.4), and 85 µM NBT. The reaction was stopped by switching off the light after 15min (four fluorescence tubes, Philips TLD/20 W, 20cm distance) and the absorbance of reduced NBT was measured at λ 560nm with a Multiskan Go Thermo Fisher Scientific UV/visible double-beam spectrophotometer.

### Structural determination of active compound

Antibacterial compound 1 obtained was then further analyzed using an ^1^H-NMR (JEOL 500 MHz), ^13^C-NMR (JEOL 125 MHz), ^1^H-^1^H COSY, and heteronuclear multiple-bond correlation (HMBC) spectrometer.

## Results

### Phytochemicals screening of the seagrass extracts

Phytochemical analysis for the presence of secondary metabolite constituents in the specimen indicated that flavonoid compounds were found in all the extracts. The methanol extract contained phenols, steroid, and tannin; the ethyl acetic extract contained phenols and steroid; whereas the water extract contained phenols and tannin, as shown in Table-2. In contrast, interestingly, no alkaloids were found in any of the extracts of both seagrass specimens.

**Table-2:** Data of phytochemical specimen extracts.

Secondary metabolites	Test method	Test result							

		MeOH		*n*-hexane		EtOAc		H_2_O	
			
		A	B	A	B	A	B	A	B
Phenolic	FeCl_3_ 5%	+	+	-	+	+	+	+	-
Flavonoid	a. HCl + Mg	-	+	-	+	-	-	-	+
	b. H_2_SO_4_ 2N	+	+	+	-	+	+	+	+
	c. NaOH 10%	+	+	+	+	+	-	+	-
Alkaloid	Dragendorff’s	-	-	-	-	-	-	-	-
Steroid	Liebermann–Burchard	+	+	-	-	+	+	-	-
Triterpenoid		-	+	-	-	-	+	-	-
Saponin	HCl + H_2_O	-	+	-	+	-	+	-	-
Tannin	FeCl_3_ 1%	+	+	-	-	-	+	+	-

### Antioxidant (SOD) activity

The increased concentration in *E. acoroides* (L.f.) Royle extract could capture free radicals, especially in the ethyl acetic extracts. Increase in antioxidant activity from *E. acoroides* (L.f.) Royle extracts of methanol, *n*-hexane, ethyl acetic, and water was dependent on the concentration of each extract. The inhibition zone values of *E. acoroides* (L.f.) Royle (SG-A) extract in these solvents were 10, 46, 7, and 33ppm, respectively, which were better or more active than those of SG-B, as shown in Table-3.

**Table-3:** Data superoxide dismutase activity of seagrass extracts.

Samples	Inhibition Activity (IC_50_/ppm)			

	*n*-hexane	EtOAc	H_2_O	MeOH
SG-A	46	7	33	10
SG-B	55	10	55	24
Quercetin	5			
Catechin	13			

### Isolation of antioxidant compound from the EtOAc of *E. acoroides* (L.f.) Royle SG-A

The active antioxidant extract of the ethyl acetate fraction (*E. acoroides* (L.f.) Royle SG-A) was selected for further separation of active compounds. Ethyl acetate extract (1.8451g) chromatographed on Silica G 60 eluted with *n*-hexane-ethyl acetate (5% stepwise) resulted in 12 fractions. Purification of fraction 6 by chromatography on ODS RP-18 eluted with H_2_O-MeOH (5% stepwise) resulted in 20 fractions. Further, purification of fractions 17 and 18 by chromatography on ODS RP-18 eluted with H_2_O-MeOH (5% stepwise) resulted in one active fraction of compound 1.

### Structure determination of compound 1

**Figure-1:**
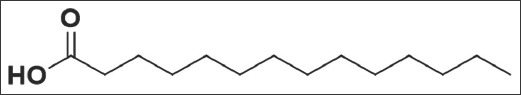
Structure of compound 1 (myristic acid or tetradecanoic acid).

Compound 1 was isolated as a yellow solid, which was soluble in methanol. The ^13^C-NMR spectrum showed 14 carbon signals, including for one carbonyl carbon at δ_C_ 173.9ppm and 13 *sp*^3^ carbons at 33.4, 31.8, 29.6, 29.5, 29.4, 29.2, 19.0, 28.8, 28.6, 28.5, 24.8, 22.4, and 13.5ppm. Correlated to protons attached to carbons, the^1^H-NMR spectrum indicated 13 methylene and one hydroxyl proton (δ_H_ 2.9ppm). From the HMBC spectrum, the carboxylic group was attached at C13 by correlation peaks between hydroxyl protons to carbon C-13 and another was attached to linear carbon. Based on the analysis of 1D and 2D-NMR spectra, the structure of compound 1 was suggested as myristic acid or tetradecanoic acid, as shown in Figure-1. Confirmation of the structure of 1 with reference to reported data indicated the synthetic compound as the same compound obtained from myristic acid as reported by Oria *et al*. [28]. Furthermore, to the best of our knowledge, the active compound 1 isolated from the seagrass *E. acoroides* (L.f.) Royle is reported for the 1^st^time in this study.

## Discussion

Recently, the need for research and development to identify a good source of marine plants that contain antioxidants has been recognized. Seagrass is one of the potential natural sources that are rich in antioxidants and the information on their bioactivity and active constituents are important aspects for improvement as new natural foods having benefits for humans.

Phytochemicals screening data presented in Table-2 give important information and show that both samples of *E. acoroides* (L.f.) Royle contain important secondary metabolites such as phenols, flavonoids, steroids, triterpenoids, saponins, and tannins, which have been reported to have anticancer, antibacterial, and antidiabetic bioactivities and act as antioxidant agents. Interestingly, alkaloid secondary metabolites were not found in both sample extracts of *E. acoroides* (L.f.) Royle. The distribution of secondary metabolites in both samples was different, which indicated that the accumulation or formation of these secondary metabolites in SG-A and SG-B seagrasses was affected by their environment. These preliminary data suggest the potential for the use of seagrass as a natural source of bioactive compounds.

To evaluate the antioxidant activity of seagrass, the extracts of SG-A and SG-B were assayed against SOD. Assay data showed that the ethyl acetate extract was the most active with an IC_50_ value of 7ppm when compared to quercetin and catechin as reference compounds that have IC_50_ values of 5 and 13ppm, respectively. The phytochemical screening and SOD assay data indicated good correlations and suggested that the high SOD activity of the EtOAc extract was derived from the phenolic and flavonoid content in the extract. The secondary metabolites of phenols, flavonoids, and steroids contained in the ethyl acetic extracts were linearly correlated with their antioxidant activity. Therefore, the initial data are an important finding for further guidance in isolating their active constituents. The ethyl acetate extract had many aromatic compounds, such as phenols and flavonoids. Aromatic compounds are chemicals that contain conjugated planar ring systems that have delocalized π-electron clouds, such as benzene and toluene [28]. Phenol is the main antioxidant found in fruit with potential health benefits that include antioxidant, anti-inflammatory, antimicrobial, and anti-carcinogenic properties [29]. Flavonoids are a large group of plant polyphenols widely distributed in nature and contained in human food. Flavonoids have attracted attention because their antioxidant activity is stronger than that of Vitamins C and E. Steroid compounds show the highest activity in the generation of intracellular ROS [30,31].

SOD is the first detoxification enzyme and the most powerful antioxidant in cells. This is an important endogenous antioxidant enzyme that acts as a component of the first line of defense against ROS. Excessive SOD can produce free radicals from the reaction (H_2_O_2_ + e^-^ →*OH). Therefore, antioxidants are needed to counteract SOD free radicals [32].

The study by Sureda *et al*. [33] showed that the antioxidant activity of the methanol extract of *Posidonia oceanica* had an IC_50_ of 7.67%; these results show slight differences from those for *E. acoroides* (L.f.) Royle extract. The antioxidant activity of the extract of *E. acoroides* (L.f.) Royle. was much better than that reported by Box *et al*. [34] from the algae *L. lallemandii* using the SOD method yielding an IC_50_ value of 14.3ppm [34]. In contrast, compound 1 showed a SOD activity of 28ppm. The SOD activity of compound 1 isolated from seagrass *E. acoroides* (L.f.) Royle is published, to the best of our knowledge, for the 1^st^time in this report.

## Conclusion

The seagrass *E. acoroides* (L.f.) Royle cultivated in two different areas showed differences in the biosynthesis of their secondary metabolites and their IC_50_ values for the SOD assay. These research data also suggest that the seagrass *E. acoroides* (L.f.) Royle has potential bioactive compounds as a source of natural antioxidants.

## Authors’ Contributions

FSIM designed the study and drafted the manuscript. MM collected the research data, UY analyzed the data. EYH reviewed the manuscript. All authors read and approved the final manuscript.
